# S93. DIETARY PATTERNS AND PHYSICAL ACTIVITY IN PEOPLE WITH SCHIZOPHRENIA

**DOI:** 10.1093/schbul/sby018.880

**Published:** 2018-04-01

**Authors:** Ane Jakobsen

**Affiliations:** Mental Health Center Copenhagen

## Abstract

**Background:**

People with severe mental disorders die 10–25 years earlier than people in the Western background population, mainly due to lifestyle related diseases, with cardiovascular disease (CVD) being the most frequent cause of death. Major contributors to this excess morbidity and mortality are unhealthy lifestyle factors including tobacco smoking, unhealthy eating habits and lower levels of physical activity. The aim of this study was to investigate the dietary habits and levels of physical activity in people with schizophrenia spectrum disorders and overweight and to compare the results with the current recommendations and with results from the general Danish population.

**Methods:**

We interviewed a sample of 428 people with schizophrenia spectrum disorders and increased waist circumference enrolled in the CHANGE trial using a Food Frequency Questionaire (FFQ) and a 24 hours recall interview, a Physical Activity Scale (PAS), scale for sssessment of positive and negative symptoms (SAPS and SANS, respectively), Brief Assessment of Cognition in Schizophrenia (BACS) and Global Assessment of Functioning (GAF). We compared with information on dietary intake and physical activity in the general Danish population from the Danish National Survey of Dietary Habits and Physical Activity in 2011–2013 (DANSDA).

**Results:**

The CHANGE participants reported a very low energy intake and their distribution of nutrients (i.e. fat, protein and carbohydrates) harmonized with the recommendations from the Danish Health Authorities, and were similar to the latest report on the dietary habits in the Danish general population. However, the intake of saturated fat, sugar and alcohol exceed the recommended amounts and the corresponding intake in the general population. The intake of fiber, vegetables and fruit and fish were insufficient and also less than in the general population. The overall estimated quality of the dietary habits was poor, only 10.7% of the participants had healthy dietary patterns, and the quality was poorer than in the general population. Even with a very liberal definition of the term “homecooked”, only 62% of the participants had taken any part in the preparation of their food. The level of physical activity was low and only one fifth of the participants complied with the recommendations of min. 30 minutes daily moderate-to-vigorous activity. Half of the CHANGE participants were smokers, compared to 17% in the general population. Negative symptoms were significantly associated with poorer dietary quality and less physical activity, whereas no such significant associations were found for cognition, positive symptoms or antipsychotic medication.

**Discussion:**

Even when accounting for some error from recall - and social desirability bias, the findings point in the direction that the average energy intake in obese people with schizophrenia spectrum disorders is not exceeding that of the general population, and that overweight may to some degree be a result of physical inactivity and metabolic adverse effects of antipsychotic medication. The physical activity level is low and the rate of tobacco smoking is high, and our results suggest that negative symptoms play a significant role. Future research should focus on bringing about lifestyle changes in this fragile population in order to reduce the excess risk of CVD and mortality.

